# The Book World of Medicine and Science

**Published:** 1899-05-06

**Authors:** 


### The Book World of Medicine and Science.

A System of Medicine by Many Writers. Edited by Thomas Clifford Allbutt, M.A., M.D., LL.D., F.R.C.P., F.R.S. Volume VI. (London: Macmillan and Co. 1899. Price 25s. net.)

First Notice.

This, the sixth volume of Allbutt's system of medicine, contains the conclusion of the section dealing with diseases of the circulatory system, and the commencement of that dealing with diseases of the nervous system. Take it altogether it is a volume which, more than its fellows, is devoted to diseases which are either rare or of only modern differentiation. Thus we find articles on right-sided valvular disease, on diseases of the lymphatic system, on Thomsen's disease, on various neurotrophic affections, on adiposis dolorosa, on erythromelalgia, and articles which deal with the most recent investigations on the subject of diseases of the spine.

The most interesting point in Dr. Newton Pitt's article on right-sided valvular lesions is the emphasis laid on tricuspid stenosis. This lesion is, of course, very rare, being far less frequently met with than tricuspid incompetence; still, it is much more common than any lesion of the pulmonary valves. Both Niemeyer and Skoda state that they had never met with an example, and were only familiar with it through museum specimens. Flint also speaks of it as a rarity, yet Dr. Pitt has been able, from the records of a single hospital, to collect 87 fatal cases during a period of 26 years out of 12,000 necropsies. Among the associated valvular lesions the most frequent was mitral stenosis, a condition which occurred in all the cases except two, and it was found that the stenosis of the mitral was far more severe than that of the tricuspid. The difficulty of diagnosing the condition may be realised when it is pointed out that in 20 of the cases analysed—that is in nearly a quarter of the whole—no bruits were specially noted in the tricuspid area in the region of the ensiform cartilage, and in only 10 cases out of the whole series was a presystolic or mid-diastolic bruit heard in the tricuspid region. The only criticism which we feel called upon to make in regard to this article is as to the conclusion drawn by the author to the effect "that, almost without exception, this stenosis is the sequel of preceding stenosis of the mitral orifice." In none of the facts related do we see anything to warrant such a conclusion, while if, as is stated, "rheumatism dominates the lesion to the exclusion of almost every other cause," it is not difficult to see that, granted a concurrent implication of both sides of the heart, the stenosis of the tricuspid would ensure exactly those physical conditions which would lead to the resulting changes in the mitral valve taking on the form of stenosis.

The article by Sir R. Douglas Powell on angina pectoris is interesting, not merely as giving a striking picture of the disease as met with in practice, but from the careful way in which the relations between angina and pseudo-angina are worked out. According to Sir Douglas Powell angina pectoris manifests itself in three forms. (1) In the first group are to be found those cases in which the disease is a pure neurosis of the cardio-vascular system, a disturbance of the innervation of the systemic or pulmonary vessels, including sometimes the vessels of the heart itself, causing their spasmodic contraction and thus increasing the peripheral resistance to circulation. The sudden excessive demand upon the propelling power of the heart thus occasioned produces a more or less painful embarrassment of its action; and in some cases this embarrassment is increased by anæmia of the cardiac muscle, due to the coincident contraction of the coronary vessels. This variety may be spoken of as angina pectoris vaso-motoria. A peculiar desire for air is generally a marked feature in these cases, the restlessness and tendency to seek fresh air by personal effort being very different from the still attitude of the subject of the graver form of angina. (2) In another and graver class of cases there is precisely the same mechanism of disturbed cardio-vascular innervation, but associated with it a diseased heart—a heart with a texturally damaged heart muscle, with a valvular defect, or indeed with both combined—and this may be designated as secondary cardiac angina. (3) In a third group of cases there are a few tolerably well-defined forms of disease of the heart in which the organ itself is the primary seat of the painful and often fatal symptoms. In these cases again the cardiac lesion may be valvular or textural. This third group may be distinguished by the designation primary cardiac angina. It includes certain cases of obstructive valvular disease, especially cases of aortic or mitral narrowing, cases of textural degeneration and ischæmia of heart, generally dependent on coronary narrowing; and lastly, although somewhat loosely, many cases of fatal syncope dependent upon degenerated heart which are unattended with other anginal phenomena. According to this conception of the matter, then, we have to look upon the anginal symptoms as due to the fact that fatigue of the cardiac as of other muscles tends to the production of cramp, and that while this condition of fatigue, or over-work (in relation to blood supply) may be due, as in the case of angina pectoris vaso-motoria, to increase of arterial tension, and in cases of secondary cardiac angina to such variations playing upon a diseased heart, it may in the third set of cases—those of primary cardiac angina—be due to conditions of the heart itself, either of muscle or blood supply, by which the ordinary demands of the economy fail to be met. In all cases, however, the actual symptoms are probably due to the fact that cramp or spastic contraction of muscle may be caused not only by fatigue, but by restricted blood supply. Indeed, in cases of fatigue the actual cause of the cramp may be that the blood supply is restricted in relation to the work to be done. Thus the factors in the production of an attack of angina "are probably the same as in those of cramp in the leg muscles, namely, restricted irrigation of the muscle through narrowing of its arterial supply, impaired removal of waste products, cramp or paralysis; but the muscle involved is in the centre of life, and its temporary disorder is apt to have a fatal issue." Diseases of the mediastinum are dealt with in an article by Dr. F. T. Roberts, which is well worth perusal. In attempting to arrive at a diagnosis in regard to the diseases of this region, it is essential not to forget how varied are the morbid conditions which may be met with. Next comes an admirable group of articles on the general diseases of the blood vessels: Thrombosis and embolism, by Professor Welch; phlebitis, by Mr. Clutton; and arterial degenerations and diseases, by Dr. F. W. Mott; followed by one on diseases of the lymphatic vessels, by Dr. Rolleston. The more specialised subject of aneurism of the aorta is dealt with by Professor Sir W. Gairdner, and that of aneurysms of arteries of the abdomen by Dr. Rolleston. All these are very well done, but we would give especial prominence to the articles by Professor Welch and Dr. Mott as giving the key to much of the pathology of diseases of the vessels, and indeed to that of many so-called diseases of other organs, which are often far more largely due to morbid conditions of their vascular supply than some people are apt to think. We will return to the consideration of this interesting volume at a later date.

